# Immunomodulator expression in trophoblasts from the feline immunodeficiency virus (FIV)-infected cat

**DOI:** 10.1186/1743-422X-8-336

**Published:** 2011-07-05

**Authors:** Veronica L Scott, Leslie A Shack, Jeffrey B Eells, Peter L Ryan, Janet R Donaldson, Karen S Coats

**Affiliations:** 1 Department of Biological Sciences, Mississippi State University, Mississippi State, MS 39762, USA; 2 Department of Basic Sciences, College of Veterinary Medicine, Mississippi State University, Mississippi State, MS 39762, USA; 3 Department of Pathobiology and Population Medicine, Mississippi State University, Mississippi State, MS 39762, USA

## Abstract

**Background:**

FIV infection frequently compromises pregnancy under experimental conditions and is accompanied by aberrant expression of some placental cytokines. Trophoblasts produce numerous immunomodulators that play a role in placental development and pregnancy maintenance. We hypothesized that FIV infection may cause dysregulation of trophoblast immunomodulator expression, and aberrant expression of these molecules may potentiate inflammation and compromise pregnancy. The purpose of this project was to evaluate the expression of representative pro-(TNF-α, IFN-γ, IL-1β, IL-2, IL-6, IL-12p35, IL-12p40, IL-18, and GM-CSF) and anti-inflammatory cytokines (IL-4, IL-5, and IL-10); CD134, a secondary co-stimulatory molecule expressed on activated T cells (FIV primary receptor); the chemokine receptor CXCR4 (FIV co-receptor); SDF-1α, the chemokine ligand to CXCR4; and FIV gag in trophoblasts from early-and late-term pregnancy.

**Methods:**

We used an anti-cytokeratin antibody in immunohistochemistry to identify trophoblasts selectively, collected these cells using laser capture microdissection, and extracted total RNA from the captured cell populations. Real time, reverse transcription-PCR was used to quantify gene expression.

**Results:**

We detected IL-4, IL-5, IL-6, IL-1β, IL-12p35, IL-12p40, and CXCR4 in trophoblasts from early-and late-term pregnancy. Expression of cytokines increased from early to late pregnancy in normal tissues. A clear, pro-inflammatory microenvironment was not evident in trophoblasts from FIV-infected queens at either stage of pregnancy. Reproductive failure was accompanied by down-regulation of both pro-and anti-inflammatory cytokines. CD134 was not detected in trophoblasts, and FIV gag was detected in only one of ten trophoblast specimens collected from FIV-infected queens.

**Conclusion:**

Feline trophoblasts express an array of pro-and anti-inflammatory immunomodulators whose expression increases from early to late pregnancy in normal tissues. Non-viable pregnancies were associated with decreased expression of immunomodulators which regulate trophoblast invasion in other species. The detection of FIV RNA in trophoblasts was rare, suggesting that the high rate of reproductive failure in FIV-infected queens was not a direct result of viral replication in trophoblasts. The influence of placental immune cells on trophoblast function and pregnancy maintenance in the FIV-infected cat requires additional study.

## Introduction

Precisely-regulated placental and decidual immunomodulatory molecules such as cytokines and chemokines are necessary to establish and maintain pregnancy [1-6]. While many of the immunomodulators expressed at the maternal-fetal interface are products of decidual leukocytes such as T cells, NK cells, dendritic cells, and macrophages [2,5,7], leukocytes are not exclusive producers of these molecules. Trophoblasts, including both cytotrophoblasts and syncytiotrophoblasts, are fetal-derived cells of epithelial origin that form the placental chorionic villi. These cells contact and attach to the maternal endometrium and serve as a barrier between maternal and fetal blood supplies in human pregnancy [8]. Trophoblasts are a source of many cytokines and other immunomodulatory molecules that support both placental development and parturition [9-11].

Mother-to-child transmission of HIV remains an important source of pediatric infection worldwide [12]. The role that HIV-infected trophoblasts play in vertical transmission and pregnancy outcome is incompletely understood. In *in vitro *experiments using trophoblastic cell lines, cells were efficiently infected with cell associated virus [13], but not cell free virus [14] due to differences in entry pathways into the cell. In the latter case, virus production was marginally increased by treatment with TNF-α and IL-1β. HIV infections can dysregulate cytokine expression in trophoblasts [15,16]. It has been speculated that aberrant expression of cytokines results in placental inflammation, facilitating transfer of the virus across inflamed membranes to infect the fetus [16].

Using the FIV-infected cat model for lentivirus vertical transmission, we demonstrated frequent reproductive failure in litters delivered at early-or late-gestation by cesarean section. Placental and fetal infection occurred in nearly all pregnancies in FIV-infected queens [17,18]. We recently reported that FIV-infection during early-and late-term gestation affects immunomodulator expression, producing a pro-inflammatory microenvironment at early, but not late pregnancy [19]. However, the specific cell populations expressing the targeted immunomodulators were not identified. In the present study, we hypothesized that FIV infection may cause dysregulation of immunomodulator expression in trophoblasts and contribute to reproductive failure. Our objectives were to determine whether feline trophoblasts microdissected from early-and late-term placentas of infected queens were infected with FIV, to quantify trophoblast expression of representative pro-and anti-inflammatory cytokines and other immunomodulators known to be important in pregnancy, and to assess whether gene expression was related to pregnancy outcome.

## Methods

### Animals and virus

All procedures utilizing cats (*Felis domesticus*) were performed with approval of the Institutional Animal Care and Use Committee of Mississippi State University. As previously reported [17,18], cats were reproductively mature, specific-pathogen-free (SPF) animals of less than 12 months of age when obtained from a commercial cattery. Ten cats were inoculated intravenously with 1 ml of a feline plasma pool containing approximately 1.3 × 10^4 ^copies/ml of FIV-B-2542 (provided by Dr. Edward Hoover) [20]. Ten cats were uninoculated and served as normal controls. Infection was confirmed within 6 weeks p.i. by detection of FIV provirus by PCR and for seroconversion using the PetChek^® ^FIV Antibody Test Kit (IDEXX Laboratories, Inc., Westbrook, ME), then queens were allowed to breed naturally with SPF toms. Toms used to breed uninfected females were never exposed to infected females and vice versa. Breeding was observed and pregnancy was confirmed by palpation and ultrasonography.

Kittens were delivered by cesarean section during week 3 gestation (early-term)[18] and week 8 gestation (late-term) [17]. Placentas were collected under sterile conditions using the following dissection procedure. The uteri were removed and individual gestational sacs were collected. Following rinses with sterile PBS, sacs were incised with a sterile scalpel, and fetuses and placentas were collected. Placental tissues were snap frozen in liquid nitrogen and cryopreserved at -80°C. The effect of FIV infection on fetal viability at early-and late-term pregnancy was previously reported [17,18]. For this study, we evaluated placental samples from both early-and late-term gestation, including placentas from viable and non-viable pregnancies. The placental tissues used in this study are listed in Table 1.

**Table 1:** Placental Tissue Evaluated in Study

Queen number and placenta	Pregnancy term	Queen FIV status	Fetal viability	Fetus FIV **Status **[17,27]	**Placenta FIV status **[17,27]	Trophoblast FIV Status/Ct value
2779 Placenta B	Early	-	Viable	NT	NT	-
2779 Placenta D	Early	-	Viable	NT	NT	-
2779 Placenta E	Early	-	Non-viable; resorption	NT	NT	-
0326 Placenta A	Early	+	Viable	+	+	-
8035 Placenta B	Early	+	Non-viable; arrested	-	+	-
8035 Placenta C	Early	+	Non-viable; arrested	+	+	-
0866 Placenta A	Early	+	Viable	+	+	-
0866 Placenta B	Early	+	Non-viable; resorption	+	+	-
9581 Placenta A	Late	-	Viable	NT	NT	-
9581 Placenta B	Late	-	Viable	NT	NT	-
9746 Placenta C	Late	-	Viable	NT	NT	-
9730 Placenta B	Late	+	Viable	+	+	-
9730 Placenta R1	Late	+	Non-viable; resorption	-	NT	-
9730 Placenta R3	Late	+	Non-viable; resorption	-	+	+/ > 37
13226 Placenta C	Late	+	Non-viable; arrested	+	+	-
13226 Placenta D	Late	+	Non-viable; arrested	+	+	-

NT = not tested

### Immunohistochemistry (IHC) to detect trophoblasts

All IHC procedures were performed using RNase-free, sterile conditions according to established protocol using polyclonal antibody to cytokeratin to label trophoblasts [21]. To enhance the capture of trophoblasts by laser capture microdissection (LCM), sections were dehydrated in 75%, 95%, and 100% ethanol. Sections were then placed in undiluted xylene until LCM was performed.

### Laser capture microdissection to capture trophoblasts

Following immunohistochemical detection in placental specimens, trophoblasts were microdissected using the Veritas™ Microdissection Instrument (Arcturus Bioscience, Inc., MountainView, CA). Microdissection was performed using the Capture IR laser (Power: 70 mW; Pulse: 2500 μsec; and Intensity: 200 mV) and UV Cutting laser (Laser spot size: ~8.0 μm and Laser power: 7.0 mW). Similar-sized fields of ~2.00 mm^2 ^were collected onto CapSure Macro LCM Caps (Arcturus). All sections and capture areas were photographed using the dissection instrument.

### RNA purification and quantification from microdissected tissues

RNA was extracted from microdissected trophoblasts of FIV-B-2542-infected and uninfected queens at early gestation (n = 5 and n = 3, respectively) and late gestation (n = 5 and n = 3, respectively), using the PicoPure RNA Isolation Kit (Arcturus). All RNA samples were treated with DNase to remove contaminating genomic DNA. RNA was quantified using the NanoDrop™ 3300 Fluorospectrometer (Thermo Fisher Scientific, Inc. Waltham, MA). All RNA was stored at -80°C until used.

### Reverse transcription-PCR to generate cDNA

Using the following cycling program: 25°C, 10 min; 37°C, 120 min; 85°C, 5 sec; 4°C, hold, cDNA was synthesized using the High Capacity cDNA Reverse Transcription Kit (Applied Biosystems, Inc., Foster City, CA) according to the manufacturer's instructions. Briefly, each reverse transcription reaction was performed in a final reaction volume of 20 μl containing 2 μl of the 10X RT buffer, 2 μl RT random primers, 0.8 μl of 25X dNTP, 1 μl of Multiscribe Reverse Transcriptase, 1 μl RNase inhibitor, 3.2 μl of RNase-free water, and 10 μl of 30-800 pg RNA template. All cDNA was stored at -20°C until used.

### Primer design of feline immunomodulators and internal control

The immunomodulators evaluated were representative pro-(TNF-β, IFN-γ, IL-1β, IL-2, IL-6, IL-12p35, IL-12p40, IL-18, and GM-CSF) and anti-inflammatory (IL-4, IL-5, and IL-10) cytokines; CD134, a secondary co-stimulatory molecule expressed on activated T cells (FIV primary receptor); CXCR4, a chemokine receptor (FIV co-receptor); and SDF-1α, the chemokine ligand to CXCR4. All the immunomodulators were known to be expressed in placental/decidual tissues and important to the establishment and maintenance of pregnancy. Sequences of PCR primers and TaqMan probes specific for feline immunomodulators and the housekeeping gene β-actin were either designed as previously reported [19,22] or obtained from the literature [17,23-26]. The primer/probe sequences are listed in Table 2.

**Table 2:** Primers and Probes used to Evaluate Immunomodulator and Virus Expression

Gene	Primer	Primer Sequence (5' to 3')	Primer Length	Probe Sequence (5' to 3')	Accession # or Reference
β actin	Forward	GACTACCTCATGAAGATCCTCACG	24	ACAGTTTCACCACCACCGCCGAGC	AB051104
	Reverse	CCTTGATGTCACGCACAATTTCC	23		
IL-1β	Forward	ATTGTGGCTATGGAGAAACTGAAG	24	TTTGCCTGCTCACAACCCCTCCAG	M92060
	Reverse	TCTTCTTCAAAGATGCAGCAAAAG	24		
IL-6	Forward	GTGTGACAACTATAACAAATGTGAGG	26	CAAGGAGGCACTGGCAGAAAACAACCT	L16914
	Reverse	GTCTCCTGATTGAACCCAGATTG	23		
IL-10	Forward	ACTTTCTTTCAAACCAAGGACGAG	24	TCTCGGACAAGGCTTGGCAACCCA	AF060520
	Reverse	GGCATCACCTCCTCCAAATAAAAC	24		
IL-12p35	Forward	ACACCAAGCCCAGGAATGTTC	21	AACCACTCCCAAACCCTGCTGCGA	U83185
	Reverse	TGGCCTTCTGAAGCGTGTTG	20		
IL-12p40	Forward	GAAGTACACAGTGGAGTGTCAGG	23	CAGTGCCTGCCCGGCTGCCG	U83184
	Reverse	GGTTTGATGATGTCCCTGATGAAG	24		
CD134	Forward	CAGGTTATGGGATGGAGAGTCG	22	TGACCAGGACACCAAGTGCCTCCAGTG	AY738589
	Reverse	TGCAAGGCTCGTAGTTCACG	20		
CXCR4	Forward	AAGGCAGTCCATGTCATCTACAC	23	ACCTCTACAGCAGTGTCCTCATCCTGGC	AJ009816
	Reverse	AGACCACCTTTTCAGCCAACAG	22		
SDF-1α	Forward	GCTACAGATGTCCTTGCCGATTC	23	TCGAGAGCCACGTTGCCAGAGCCA	AB011965
	Reverse	TCTTCAGCCTCGCCACGATC	20		
IL-2	Forward	ACGGTTGCTTTTGAATGGAG	20	CCCCAAACTCTCCAGGATGCTCA	Ref 23
	Reverse	CAATTCTGTGGCCTTCTTGG	20		
IL-4	Forward	CCCCTAAGAACACAAGTGACAAG	23	TTCTGCAGAGCCACAACCGTGC	Ref 23
	Reverse	CCTTTGAGGAATTTGGTGGAG	21		
IL-5	Forward	TGCTTCTGCATTTGAGTTTG	20	TGGCAGAAACATAGGCAGCCCC	Ref 23
	Reverse	CAGCCTATTCATGGGACTTTG	21		
IL-18	Forward	GGAGATCAACCTGTGTTTGAGGAT	24	ATTCTGACTGTACAGATAATGCACCCCGGAC	Ref 25
	Reverse	GATGGTTACTGCCAGACCTCTAGTG	25		
GM-CSF	Forward	AATGAAACGGTAGAAGTCGTCTCTG	25	TTGACCCTGAGGAGCCGAATTGCC	Ref 26
	Reverse	CGTACAGCTTTAGGTGAGTCTGCA	24		
IFN-γ	Forward	TGGTGGGTCGCTTTTCGTAG	20	CATTTTGAAGAACTGGAAAGAGGAGAGTGATAAAACAAT	Ref 24
	Reverse	GAAGGAGACAATTTGGCTTTGAA	23		
TNF-α	Forward	CTTCTCGAACTCCGAGTGACAAG	23	TAGCCCATGTAGTAGCAAACCCCGAAGC	Ref 26
	Reverse	CCACTGGAGTTGCCCTTCA	19		
GAG	Forward	GTATGATCGTACTCATCCTCCTGAT	25	AGACCACTGCCCTACTTCACTGCCG	Ref 17
	Reverse	TCTACATTGCATTCTGGCTGGT	22		

### Evaluation of expression of immunomodulators using TaqMan real-time PCR

The real-time PCR (qPCR) used an iCycler (Bio-Rad) and the following cycling program: 50°C, 2 min; 95°C, 10 min; 60 X (95°C, 15 s; 60°C, 1 min). Each reaction contained 10 μl of the 2X TaqMan^® ^Universal PCR Master Mix (Applied Biosystems, Life Technologies Corp., Carlsbad, CA), 1 μl of forward and reverse primers (10 pmol/μl), 1 μl of the respective probe (100 fmol/μl), and ~2-4 μg cDNA. For every placental cDNA sample, parallel reactions were performed in triplicate simplex reactions on separate plates for each gene. Due to the low concentration of RNA isolated from extracted cells, and consequently, the limited amount of cDNA available for use in qPCR, standard curves for internal control and target gene amplicons were generated using pooled, serially-diluted whole placental tissue RNA from representative uninfected cats. Each titration simplex reaction contained 6.25 μl of the 2X Thermoscript™ reaction mix, 0.25 μl of Thermoscript™ RT/Platinum^® ^*Taq *Mix (Invitrogen), 1 μl of forward and reverse primers (10 pmol/μl), 1 μl of the respective probe (100 fmol/μl), and five-fold diluted 2 μg RNA as starting titration template. The standard curves were used to normalize for differences in PCR efficiency between the internal control and target genes and between sample plate runs. Differences in the amount of template cDNA in each reaction was corrected by the cycle threshold (Ct) value for β-actin. Normalized samples were divided by the calibration, generating the relative expression levels.

### Statistical analysis

Statistical evaluation of fetal viability from early-and late-term gestation was previously reported [17,18]. Statistical analyses of immunomodulator expression for early-and late-term control and FIV-B-2542-infected trophoblasts were done using single-factor ANOVA and the two independent sample Wilcoxon rank sum test (SOCR Analysis, University of California, Los Angeles, CA).

## Result

### Microdissection of trophoblasts from placental tissues

**Figure 1:**
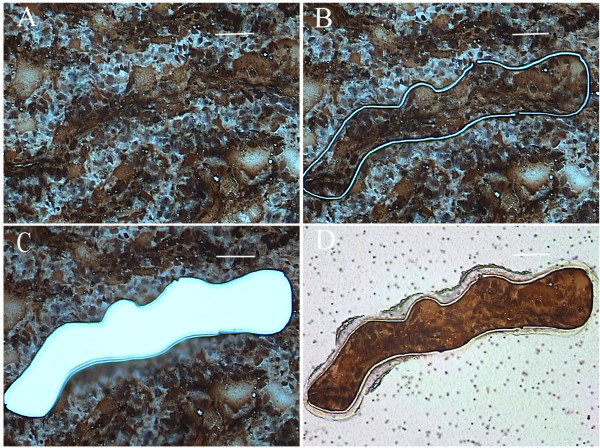
**Laser Capture Microdissection of IHC-stained Trophoblasts from a Representative FIV-negative Cat**. Trophoblasts were labeled using antibody to cytokeratin, and cytokeratin-positive trophoblasts were evident by brown staining. (A) Trophoblasts were selectively identified. (B) Areas of trophoblast-rich populations were cut and captured using the UV cutting laser and Capture IR laser, respectively. (C) Selected trophoblasts were removed from the tissue. (D) Trophoblasts were attached to the cap. All photography was performed with the 20X objective. Bars = 200 microns.

Figure 1 shows a representative placental specimen following immunolabeling with anti-cytokeratin antibody and laser capture microdissection of trophoblasts. Cytokeratin labeling was specific for trophoblasts [21].

### Expression of FIV gag in trophoblasts

FIV gag was detected in only a single, late-term trophoblast specimen, collected from a placenta associated with a fetal resorption (Table 1). The high Ct value (greater than 37) indicated low level infection of this trophoblast specimen. FIV gag was previously detected in RNA from whole placental tissues using the same protocol [27].

### Expression of immunomodulators in trophoblasts

Messenger RNA for IL-4, IL-5, IL-6, IL-1β, IL-12p35, IL-12p40, and CXCR4 was expressed in all early-and late-term trophoblasts. Messenger RNA for TNF-α, IFN-γ, IL-2, IL-18, IL-10, GM-CSF, CD134, and SDF-1α was not detected in trophoblasts from either stage of pregnancy. All immunomodulators were amplified from RNA extracted from whole placental tissues used for generating standard curves (data not shown), indicating that failure to detect some of them in trophoblasts was not a result of inadequate qPCR conditions.

**Figure 2:**
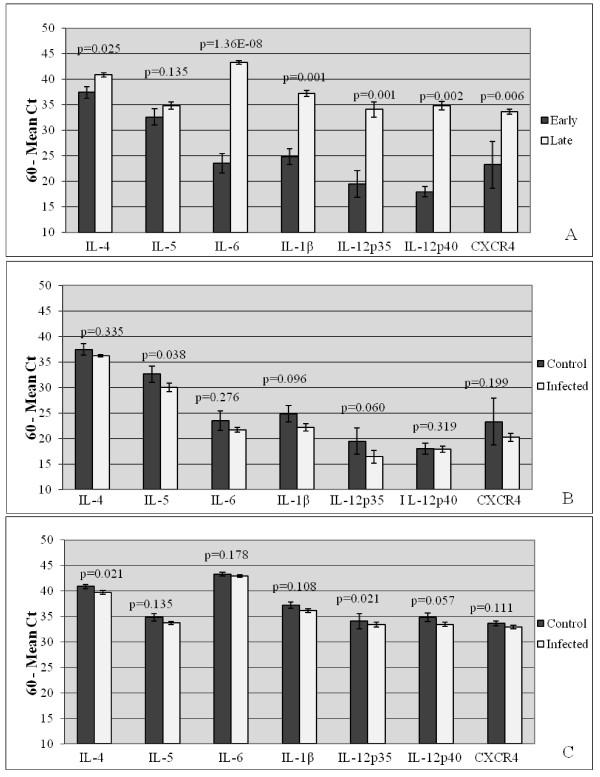
**Relative Expression of Immunomodulators in Trophoblasts from Early-and Late-Term Feline Placentas**. Real-time RT-PCR analysis of feline placental trophoblast expression of the pro-inflammatory cytokines (IL-6, IL-1β, IL-12p35, and IL-12p40), the anti-inflammatory cytokines (IL-4 and IL-5), and the chemokine receptor (CXCR4) in: (A) early-term uninfected placentas (n = 3) versus late-term uninfected placentas (n = 3); (B) early-term FIV-infected (n = 5) versus uninfected placentas; (C) late-term FIV-infected (n = 5) versus uninfected placentas. Bars represent mean Ct values substracted from a negative endpoint (60-mean Ct), bracketed by standard errors of the mean. P values obtained from single factor ANOVA and Wilcoxon rank sum test are noted. P values ≤ 0.05 were considered significant.

To determine normal feline trophoblast expression of the detected immunomodulators between the two stages of pregnancy, data obtained from control placentas from early-and late-term were compared. Trophoblasts from control cats at late gestation expressed more IL-4, IL-6, IL-1β, IL-12p35, IL-12p40, and CXCR4 mRNA than trophoblasts from control cats at early gestation (p < 0.05). Only IL-5 did not differ between the two stages of pregnancy (p = 0.135) (Figure 2A).

To determine the effect of FIV infection on expression of these immunomodulators, trophoblasts from FIV-infected and control queens at both early-and late-term gestation were compared. At early gestation, the marginally-reduced expression of nearly all immunomodulators in FIV-infected cats did not reach the level of significance. The expression of the anti-inflammatory cytokine IL-5 alone was decreased to a significant level (p = 0.038) (Figure 2B). At late gestation, FIV-infection resulted in decreased expression of the anti-inflammatory cytokine IL-4 (p = 0.021) and the pro-inflammatory cytokine IL-12p35 (p = 0.021); decreased expression of the pro-inflammatory cytokine IL-12p40 (p = 0.057) approached, but did not reach significance. There was no significant effect on the expression of the remaining immunomodulators (Figure 2C).

**Figure 3:**
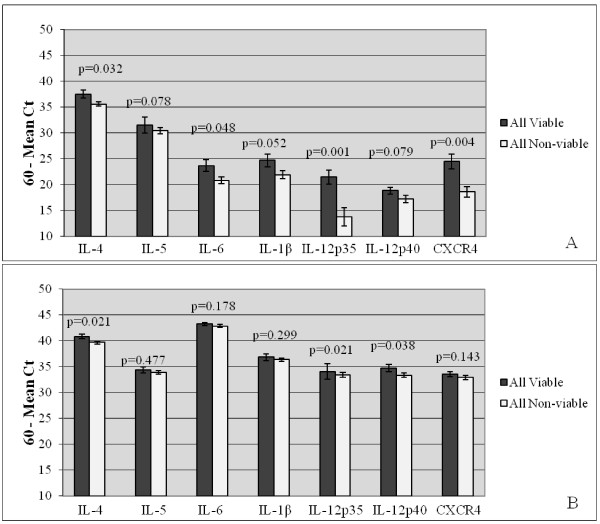
**Relative Expression of Immunomodulators in Trophoblasts from Early-and Late-Term Feline Placentas from Viable and Non-viable Pregnancies**. Real-time RT-PCR analysis of trophoblast expression of the pro-inflammatory cytokines (IL-6, IL-1β, IL-12p35, and IL-12p40), the anti-inflammatory cytokines (IL-4 and IL-5), and the chemokine receptor (CXCR4) in early-(A) and late-term (B) placentas from all viable (n = 4 each for early and late tissues) versus all non-viable (n = 4 each for early and late tissues) pregnancies. Bars represent mean Ct values substracted from a negative endpoint (60-mean Ct), bracketed by standard errors of the mean. P values obtained from Wilcoxon rank sum test are noted. P values ≤ 0.05 were considered significant.

To determine whether cytokine expression was related to fetal viability regardless of infection, trophoblasts from all viable pregnancies were compared to trophoblasts from all non-viable pregnancies for both early-and late-term gestation (Figure 3). At early gestation, trophoblasts from non-viable pregnancies expressed significantly less IL-4 (p = 0.032), IL-6 (p = 0.048), IL-12p35 (p = 0.001), and CXCR4 (p = 0.004) mRNA than those of viable pregnancies (Figure 3A). The decreased IL-1β expression in non-viable pregnancies approached, but did not reach the level of significance (p = 0.052). At late gestation, trophoblasts from non-viable pregnancies expressed significantly less IL-4 (p = 0.021), IL-12p35 (p = 0.021), and IL-12p40 (p = 0.038) than trophoblasts from viable pregnancies (Figure 3B).

## Discussion

Using the FIV-infected, pregnant cat to evaluate lentivirus-induced placental immunopathology and pregnancy outcome, we and others reported vertical transfer of FIV-B-2542 at early-and late-term pregnancy [17,18,28], with more than 50% of offspring infected. We found fetal non-viability to be significantly higher in FIV-infected queens at both stages of pregnancy [17,18]. While the mechanism of FIV-induced reproductive failure is unresolved, placental inflammation appears to accompany FIV infection, especially at early pregnancy. In a previous report by our laboratory which evaluated immunomodulator expression in whole placental specimens [19], an FIV-induced, pro-inflammatory microenvironment at early, but not late pregnancy, was evident, including increased expression of IL-6 and IL-12, decreased expression of IL-10, and IL-12:IL-10 ratios that favored the pro-inflammatory cytokine in infected cats at early pregnancy.

In the present study, the effect of FIV infection on immunomodulator expression in a targeted cell population, trophoblasts, was evaluated at early and late gestation. Immunomodulators produced by trophoblasts play important roles in the progression of pregnancy. Thus, understanding how infections or perturbed pregnancy correspond to altered gene expression specifically in trophoblasts, *in situ *and *in vitro*, has been the focus of many investigations [29-32]. We were able to detect expression of IL-4, IL-5, IL-6, IL-1β, IL-12p35, IL-12p40, and CXCR4 in feline placental trophoblasts at both time points during feline gestation. The relative expression of all immunomodulators increased in normal specimens as gestation progressed, indicating increased immunomodulatory function of feline trophoblasts at late pregnancy. At early gestation, FIV infection was associated with slightly decreased expression of all cytokines, although only the expression of IL-5 (an anti-inflammatory cytokine) decreased to significant levels. At late gestation one anti-inflammatory cytokine (IL-4) and one pro-inflammatory cytokine (IL-12p35) were decreased in FIV-infected queens. The data suggest that FIV infection of the queens caused a minor effect on trophoblast function, and an FIV-induced pro-inflammatory effect in trophoblasts was not clearly evident.

Non-viable pregnancies were accompanied by decreased trophoblast expression of both pro-and anti-inflammatory immunomodulators at early-(IL-4, IL-6, IL-12p35, and CXCR4) and late-term (IL-4, IL-12p35, and IL-12p40). One would predict reduced placental function in dying pregnancies. However, decreased gene expression was not significant for all immunomodulators, suggesting that reduction in immunomodulator function was not solely attributable to deterioriation of the placenta in non-viable pregnancies. The decreased expression of IL-4 and IL-6 in early placental trophoblasts from non-viable pregnancies parallels the IL-4 and IL-6 expression patterns found in the endometrial tissue and deciduas of women with frequent implantation failures[33] and recurrent abortions [34]. IL-4 is a Th2 cytokine that is mainly involved in the peri-implantation period of placental development [35]. Along with transforming growth factor-β (TGF-β), IL-4 mediates the process of trophoblast invasion during human placentation [36-39]. IL-4 and IL-6 contribute to the angiogenesis of trophoblastic villi [40]. Silencing of IL-6 in JEG-3 cells (choriocarcinoma cells maintaining trophoblast-like characteristics) using siRNA resulted in significantly reduced proliferation of those cells [41]. IL-1β activates matrix metalloprotease 3 (MMP-3), a protease that digests extracellular matrix proteins and allows trophoblast penetration of the maternal endometrium [42]. In the present study, the decrease in expression of IL-1β approached significance (p = 0.052) at early pregnancy. In the human placenta, CXCR4, expressed by trophoblasts, is also important in trophoblast invasion via interaction with its ligand, SDF-1α, expressed on decidual stromal cells [43]. In addition, this interaction protects trophoblasts from apoptosis via stimulation of anti-apoptotic pathways [44]. IL-12, a heterodimeric cytokine consisting of 35 and 40 kd protein subunits, is also an important regulator of trophoblast invasion. It inhibits trophoblast invasion by causing an IFN-γ-dependent reduction in expression of MMP-2, MMP-9, urokinase-type plasminogen activator, and other proteases that digest extracellular matrix proteins. IL-12 also causes an increase in expression of inhibitors of these proteases [45]. The decreased expression of IL-4, IL-6, IL-12p35, and CXCR4 in trophoblasts from early-term non-viable pregnancies shows dysregulation of putative mediators of feline trophoblast invasion during early feline gestation, an outcome that could result in nutrient and oxygen deprivation of the developing fetus and account for the increased reproductive failure that occurred in infected animals.

In contrast, cultured placental trophoblasts from HIV-infected women expressed significantly higher amounts of IL-1β, IL-6, and TNF-α than placentas from uninfected women [16]. These data indicate that HIV-infection during pregnancy results in increased cytokine expression and that a pro-inflammatory microenvironment (an environment potentially conducive to vertical transfer) exists in HIV-infected placentas. We did not see this trend in trophoblasts collected from the cat model. However, the study of human tissues used cultured trophoblasts, whereas in the present study, RNA was extracted from trophoblasts microdissected from frozen placentas. Whether culturing the cells affected gene expression is unknown.

While we previously reported the detection of mRNA for the primary FIV receptor CD134 in whole feline placental tissues [46], we did not detect CD134 in any trophoblast sample from early-or late-term pregnancy. Correspondingly, FIV was detectable at a low level (Ct > 37) in only one trophoblast specimen, a placenta from a late-term fetal resorption, indicating that trophoblasts may not be highly susceptible or permissive to FIV infection. This result was surprising, given the high frequency of fetal infection that was documented in these same cats [17,18], and suggests that infected trophoblasts may not be the source of fetal infection. We speculate that immune cells in the surrounding microenvironment, such as regulatory T cells (Tregs) or Th17 cells, the former of which are known to support the replication of FIV [47,48], may influence trophoblast function and serve as the source of fetal infection. Our laboratory is currently investigating feline Tregs and Th17 cells in lentivirus-induced placental immunopathology and pregnancy failure.

A limitation of this study was the small number of tissues evaluated by LCM, particularly in terms of separating viable versus non-viable tissues. LCM is a procedure which is laborious, time-intensive, and expensive to perform, so limiting the samples collected was a necessity. We attempted to minimize inter-cat variability by evaluating multiple placentas from the same queen where possible. The differences that we have historically observed between placentas from the same queen assured us that we can consider placentas from the same queens as independent specimens [17,19,27]. Yet, the inclusion of additional placental specimens may have strengthened the statistical power and allowed us to identify additional viral effects. In addition, it is impossible to eliminate all cell contamination from the microenvironment surrounding the trophoblasts due to limitations in the precision of focusing the laser. However, our samples were highly enriched for trophoblasts, as is evident by the well-defined tissue lifts that we were able to achieve (Figure 1). LCM followed by qPCR has been used as a means to collect trophoblasts from whole placental specimens and measure gene expression in these cells by others [49]. Regardless of the limitations, this study provides novel information. Our previous reports clearly reveal that FIV infection results in significantly higher rates of reproductive failure, probably by inducing placental immunopathology [17-19]. The present data shed additional light on this anomaly, suggesting that aberrant expression of trophoblast cytokines that may regulate trophoblast invasion accompanies fetal demise.

## Conclusion

Feline trophoblasts expressed an array of pro-and anti-inflammatory immunomodulators whose expression increased from early to late pregnancy in normal tissues, while some immunomodulators that were expressed in whole placental tissues were not detected. A virus-induced, pro-inflammatory environment was not detected in trophoblasts. Non-viable pregnancies were associated with decreased expression of immunomodulators which regulate trophoblast invasion in other species, a finding which may explain the high rate of reproductive failure in FIV-infected cats. The detection of FIV RNA in trophoblasts was rare. This result suggests that reproductive failure and altered trophoblast gene expression in FIV-infected queens was not a direct result of viral infection of trophoblasts, but perhaps was influenced by cells in the microenvironment adjacent to trophoblasts. The effects of placental immune cells on trophoblast function and pregnancy maintenance in the FIV-infected cat requires additional study.

## Competing interests

The authors declare that they have no competing interests.

## Authors' contributions

VLS conducted all aspects of the research and data analysis and wrote drafts of the manuscript. LAS provided technical assistance with qPCR. JBE, PLR, and JRD served as consultants and provided important advice relative to LCM, trophoblast IHC, and cDNA synthesis, respectively. KSC secured funding, directed the research, contributed to data analysis, and finalized the manuscript. All authors read and approved the manuscript.
